# Probing Peptide and Protein Insertion in a Biomimetic S-Layer Supported Lipid Membrane Platform

**DOI:** 10.3390/ijms16022824

**Published:** 2015-01-27

**Authors:** Samar Damiati, Angelika Schrems, Eva-Kathrin Sinner, Uwe B. Sleytr, Bernhard Schuster

**Affiliations:** 1 Institute for Synthetic Bioarchitectures, Department of NanoBiotechnology, University of Natural Resources and Life Sciences, Muthgasse 11, Vienna 1190, Austria; E-Mails: sdamiati@kau.edu.sa (S.D.); angelika.schrems@gmail.com (A.S.); eva.sinner@boku.ac.at (E.-K.S.); 2 Department of Biochemistry, King Abdulaziz University, Jeddah 21465, Saudi Arabia; 3 Institute for Biophysics, Department of NanoBiotechnology, University of Natural Resources and Life Sciences, Muthgasse 11, Vienna 1190, Austria; E-Mail: uwe.sleytr@boku.ac.at

**Keywords:** biomimetics, supported lipid membrane, S-layer, planar membrane platform, membrane-active peptide and protein

## Abstract

The most important aspect of synthetic lipid membrane architectures is their ability to study functional membrane-active peptides and membrane proteins in an environment close to nature. Here, we report on the generation and performance of a biomimetic platform, the S-layer supported lipid membrane (SsLM), to investigate the structural and electrical characteristics of the membrane-active peptide gramicidin and the transmembrane protein α-hemolysin in real-time using a quartz crystal microbalance with dissipation monitoring in combination with electrochemical impedance spectroscopy. A shift in membrane resistance is caused by the interaction of α-hemolysin and gramicidin with SsLMs, even if only an attachment onto, or functional channels through the lipid membrane, respectively, are formed. Moreover, the obtained results did not indicate the formation of functional α-hemolysin pores, but evidence for functional incorporation of gramicidin into this biomimetic architecture is provided.

## 1. Introduction

Biological protein pores and channel-forming peptides provide nanoscopic pathways for the flux of ions and other charged molecules across the hydrophobic portion of membranes in a variety of cells. These functions are essential for life and serve as key components in intercellular and intracellular communications [1,2] and hence, represent very attractive drug targets. However, at the same time, studying membrane proteins and their pharmacological application potential is a challenging task since their activity is directly related to the quality and components building up the essential surrounding hydrophobic environment [3,4]. In an effort to develop synthetic membrane mimics of the natural cell membrane in both structure and function, fabrication of a supported lipid bilayer on a solid substrate is receiving growing attention [5,6,7]. Solid supported lipid membranes are a well-known class of model systems, extremely useful for studying biophysical and biochemical properties of biological membranes, their constituted lipid and protein molecules, and are highly attractive for industrial research and technological development [4,6,8]. In order to design a synthetic membrane compatible for membrane-spanning proteins that overcome challenges facing protein reconstitution, a strategy based on tether molecules or biocompatible cushions separating the lipid bilayer from the solid support has been developed [6,7]. This approach has many advantages, e.g., reduces the non-specific binding of proteins to solid substrates, increase lipid membrane stability and longevity, provides a lubricant layer allowing the lipid membrane to remain mobile and hence, may assist self-healing of local defects even when deposited on macroscopic supports, and may allow insertion of bulky transmembrane proteins into the lipid bilayer by providing space between the membrane and solid support for protruding protein domains [6,7,8,9,10].

Two-dimensional arrays of proteinaceous subunits forming surface layers on prokaryotic cells, termed S-layers, are the common surface structure of almost all archaea and most bacteria [11,12,13]. In general, S-layer proteins have the unique property that, following disruption by chemical agents from bacterial cells, monomers of the protein can reassemble to their original lattice structure in suspension, on solid surfaces, at air–water interfaces, and on phospholipid mono- and bilayers [5,14,15,16,17]. Most S-layer lattices are composed of a single protein or glycoprotein species (molecular weight (*M*w) 40–200 kDa) with the subunits aligned either in oblique, square or hexagonal lattice symmetry having a center-to-center spacing of approximately 5–30 nm. Bacterial S-layers are generally 5–10 nm thick whereas those of archaea reveal a thickness of up to 70 nm. Most S-layers show a smooth outer and a more corrugated inner surface. S-layer proteins shape highly porous lattices with identical pores of 2–8 nm in diameter and a surface porosity ranging between 30% and 70% [11,13,16,18]. In bacteria, the S-layer proteins are linked to each other and to the underlying cell envelope layer by non-covalent forces [13,17,18,19,20].

In the present study, the unique intrinsic features of the S-layer protein SbpA isolated from *Lysinibacillus sphaericus* CCM 2177 were exploited as biomimetic tethering structure to keep the lipid membrane separated from the protein-denaturing gold surface, as water-containing ion reservoir for repeatedly electrochemical experiments, as stabilizing scaffold for the lipid membrane that preserves also the fluidity of the membrane to a large extent and, because of its highly porous structure, as place holder where domains of transmembrane proteins may extend into the water-filled pores [21,22,23]. The building principle of this so-called S-layer supported lipid membrane (SsLM) is copied from the supramolecular cell envelope structure of archaea, which possess an S-layer as exclusive cell wall component external to the plasma membrane [11,17,24]. Archaea dwell under very harsh conditions [24] and for this reason it is concluded that mimicking the cell envelope structure results in very stable model lipid membranes [5,11]. Indeed, evidence has been provided that an SbpA lattice recrystallized on lipid membranes act as membrane stabilizing scaffold and induce a “nanopatterned membrane fluidity” and extended the longevity of the whole composite architecture [5,17,23].

A further important parameter determining protein or peptide incorporation, which is a functional channel or pore formation, is to choose the proper lipid composition. The performance of lipid bilayers depend largely on the physical and chemical properties of the lipid molecules [25]. Since the permeability and structural features of synthetic membranes are sensitive to their membrane components and to events occurring at the interface or within the bilayer, it is important to mention that recrystallized bacterial S-layer proteins like SbpA are not intercalating into the hydrophobic part of the membrane or decrease the membrane fluidity [26], which is critical for, e.g., ion channel functionality [27].

**Figure 1:**
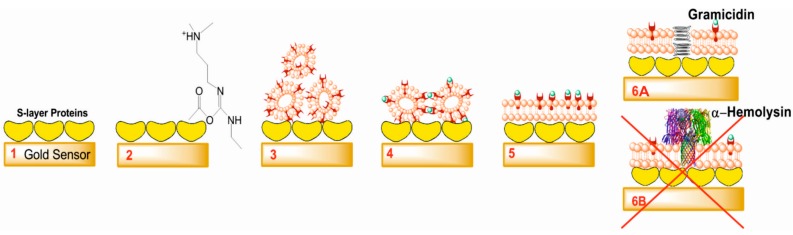
Schematic sketch (not drawn to scale) showing the step-by-step formation and functionalization of the S-layer supported lipid membrane (SsLM). (**1**) Recrystallization of the S-layer protein SbpA on the gold sensor; (**2**) Chemical activation of SbpA; (**3**) Binding of small unilamellar vesicles (SUVs) via lipid linker molecules on the SbpA lattice; (**4**) Addition of Eu^3+^-ions to form inter-liposomal complexes of SUVs mediated by the β‑diketone ligand molecules; (**5**) Formation of a lipid bilayer structure on SbpA; (**6A**) Gramicidin incorporated in an SsLM; and (**6B**) α-Hemolysin reconstituted in an SsLM (could not be achieved in the present study).

One of the major advantages of planar supported lipid bilayer platforms is the wide range of surface-sensitive techniques, which can be applied to study membrane characteristics or the protein/membrane interaction [5,7,13]. Quartz crystal microbalance with dissipation monitoring (QCM-D) as one of these techniques allows real time quantitative measurements of the mass of surface-attached material on the sensor surface including the bound/coupled liquid medium [28,29]. Moreover, the energy loss referring to information related to changes in the viscoelastic properties of the deposited layer can also be monitored [30]. Combining QCM-D with electrochemical impedance spectroscopy (EIS) provides complementary information about the electrical properties of the membrane and whether proteins or peptides have been functionally reconstituted into membranes or not. EIS monitors the integrity of lipid membranes in real time and is very sensitive to changes in membrane properties such as membrane homogeneity, thickness, and resistance [31]. In the present study, combined QCM-D and EIS measurements on one and the same preparation were used for the first time, for monitoring lipid membrane formation on the SbpA lattice and subsequent interaction with the channel-forming peptide gramicidin and the pore-forming protein α-hemolysin, respectively (Figure 1).

The pore-forming toxin α-hemolysin is an extracellular protein secreted by *Staphylococcus aureus.* The water-soluble monomeric protein is comprised of 293 amino acids with a *M*w of 33 kDa [32]. The monomers attach to the lipid membrane and form spontaneously heptameric pores with a diameter ranging from 14 to 46 Å [33]. The α-hemolysin pore has a mushroom-like shape with the stem penetrating the lipid membrane and the cap extending into the extracellular space [33,34].

The interaction of the channel-forming ionophore gramicidin with SsLMs was also investigated. Gramicidin, synthesized by *Bacillus brevis*, is a water-insoluble, linear polypeptide with a *M*w of ~1900 Da. The channel of gramicidin is built up by two antiparallel oriented monomers bound to each other by six hydrogen bonds [35]. Hence, channel formation for the transport of monovalent cations is only possible in a membrane comprising of a lipid bilayer structure [36].

The present study is motivated by the high demand for studying membrane-active peptides and membrane proteins in a biomimetic environment. This intention is of paramount importance as the results of genome mapping showed that approximately one-third of all genes of an organism encode for membrane proteins, which are key factors in cell’s metabolism and thus, in health and disease. Moreover, membrane proteins constitute preferred targets for pharmaceuticals (at present more than 60% of all consumed drugs) [3]. Planar model lipid membranes, in particular SsLMs may receive widespread recognition in drug discovery and protein–ligand screening. Moreover, combining QCM-D and EIS to monitor structural changes and the formation of functional pores in the lipid membrane has now recognized to constitute a straightforward approach to elucidate the mode of action and function of membrane-active peptides and membrane proteins [37]. Recently, it became possible for the first time to generate SsLMs by a sophisticated vesicle fusion technique without the need of an aperture [28], which is a mandatory requirement for QCM-D measurements. Moreover, the electrical sealing accomplished by the lipid bilayer is sufficient to examine ion flux even through single reconstituted membrane functions. Hence, this study constitutes the first attempt to study α-hemolysin and gramicidin incorporation in SsLMs directly generated within a QCM-D cell without the need of an aperture combined with EIS measurements on one and the same functionalized SsLM preparation.

## 2. Results and Discussion

### 2.1. Assembly of S-Layer Supported Lipid Membranes

**Figure 2:**
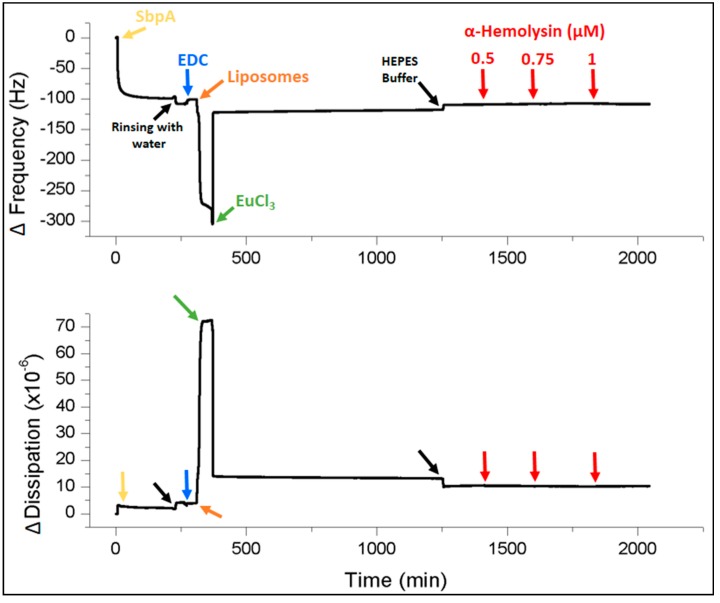
Representative shift in frequency (**bottom**) and dissipation (**top**) *versus* time during the formation of SsLMs and the subsequent addition of increasing amounts of α-hemolysin at different concentrations (0.5, 0.75 and 1 µM) as indicated in the plots. All shown QCM-D data were recorded at the 7^th^ overtone.

The QCM-D technique was not only applied to follow SsLM formation, (*i.e.*, recrystallization of SbpA on the gold-coated sensor crystal and successive formation of lipid bilayer by binding of small unilamellar vesicles (SUVs) and subsequent fusion of the latter), but was also used to investigate the incorporation features of the pore-forming protein α-hemolysin and the ionophore gramicidin. Figure 2 shows representative data indicating the change in frequency and dissipation in the course of SsLM formation and interaction with the membrane protein α-hemolysin as a function of time.

In a first step, the assembly of SbpA from solution on gold sensors (schematically depicted in Figure 1, step 1) was determined and showed a rapid decrease of the frequency level to ~−100 Hz and a slight increase of the energy dissipation level (<1 × 10^−6^) (Figure 2). Both values recorded by QCM-D for SbpA recrystallization on the gold sensor are in good agreement with previously published work [19,28,37,38,39].

Two obstacles face lipid bilayer formation onto S-layer protein lattices: vesicle attachment and their subsequent spontaneous fusion. In general, S-layer protein lattices show the characteristics that almost no biomolecules stick to them [11]. As a result, vesicles would simply roll over the proteinaceous lattice [40]. This intrinsic feature may be a biological function of the S-layer lattice covering many prokaryotic cells [5,11]. Therefore, 1-ethyl-3-(3-dimethylaminopropyl) carbodiimide hydrochloride (EDC) was used to activate the carboxyl groups on the S-layer lattice forming highly reactive *O*-acylisourea intermediates (Figure 1, step 2) [15]. Subsequently a solution of SUVs comprising of egg yolk phosphatidyl choline (Egg PC), 1,2-dimyristoyl-*sn*-glycero-3-phospho-ethanolamine (DMPE; in both monolayers of the SUVs or only in the outer one), cholesterol and β‑diketone ligand (see Experimental Section) was passed over the SbpA lattice (Figure 1, step 3). This lipid was chosen for several reasons: the Egg PC is the bulk component forming the spherical vesicle; DMPE, as linker molecule is covalently bound with its terminal amino group to the activated SbpA; cholesterol has put in because it is the primary target for α-hemolysin [41] while, however, it attenuates modestly the action of gramicidin on phospholipid bilayers [42]. The amphiphilic β-diketone ligand molecules incorporated in SUVs master vesicle fusion immediately after addition of Eu^3+^-ions (Figure 1, steps 4 and 5). The mechanism is explained by the formation of inter-vesicular Eu^3+^-ion/β-diketone ligand (1/2) complexes [43], which tear the SUVs down and open them to form a planar lipid bilayer. The dramatic structural change in the lipidic layer can nicely be investigated by QCM-D. Initially, the bound layer composed of SUVs gave rise to a huge decrease in frequency (~−300 Hz) and increase in dissipation (~75 × 10^−6^). These data correspond to a very soft, highly water–containing vesicular layer. After Eu^3+^-ion triggered vesicle fusion the frequency increased instantly to ~−120 Hz and the dissipation decreased to ~10 × 10^−6^ (Figure 2). The shift in frequency and dissipation provide clear evidence for the change of the attached lipid structure. The obtained difference in frequency between the S-layer lattice with and without the attached lipid membrane is approximately 25 Hz, which is in good agreement with the previously reported QCM‑D response observed at the formation of a bilayer lipid membrane [28,44]. In addition, the same dissipation value was previously reported for SsLMs [28,29]. Subsequently the membrane was rinsed with 150 mM NaCl, 10 mM HEPES buffer, pH 7.4, to remove unbound excess material and to perform EIS measurements.

### 2.2. Electrical Characterization of S-Layer Supported Lipid Membranes

Electrical characterization of the composite architecture was conducted by measuring the resistance and capacitance from each formed layer. The capacitance has significantly decreased after recrystallization of SbpA on bare gold surface from 30 to 22 µF·cm^−2^ and finally to ~3 µF·cm^−2^ after formation of a lipid bilayer structure. However, due to the high amount of entrapped water in the inter-membrane region,* i.e*., the S-layer lattice, one cannot assume that the membrane capacitance is equally distributed over the whole surface [45]. For this reason, the membrane capacitance was estimated by fitting the data of the impedance spectra of the lipid membrane on the S-layer lattice to a circuit containing a constant phase element (CPE) in parallel to the membrane resistance. The resistance, in contrast, increased from ~0.01 MΩ·cm^2^ for gold to ~0.55 MΩ·cm^2^ for the SbpA lattice and to 5.16 ± 1.27 MΩ·cm^2^ (*n* = 8) for the lipid bilayer since each additional layer acts as a further barrier for charged molecules with the hydrophobic part of the lipid membrane being the most prominent one [40].

### 2.3. Incorporation of Gramicidin

Addition of gramicidin at different concentrations to the preformed SsLM (Figure 1, step 6A) revealed negligible shifts in the frequency and dissipation values indicating no measurable change in the structure of the lipid membrane. Hence, QCM-D alone cannot provide occlusive evidence for functional peptide insertion into the lipid membrane. Therefore, a possible ionophore formation has been scrutinized by EIS. The membrane resistance decreased significantly from initially 4.56 to 4.12 MΩ·cm^2^ and 4.11 to 3.37 MΩ·cm^2^ before and upon addition of 1, 2.5 and finally 5 µM gramicidin, respectively. The significant decrease in membrane resistance is a result of gramicidin incorporation and subsequent channel formation. In contrast, the membrane capacitance remained constant and showed no significant change. It has been proven that gramicidin channels lack any gating mechanism as it forms conducting dimers when the peptide is transferred to both leaflets of the bilayer [1,35]. In previous studies, evidence for the functional incorporation of gramicidin, but also valinomycin and alamethicin, into lipid membranes resting on an SbpA layer deposited on an ultrafiltration membrane has been provided [22,40]. Moreover, SsLM generated by the “rapid solvent exchange technique” has been used to study a negatively charged analogue of the antimicrobial peptide peptidyl-glycine-leucine-carboxyamide (PGLa(−)). In this study, not only successful incorporation of PGLa(−) and its mode of action could be determined, it was even possible to deduce the formation of toroidal pores [37].

Since gramicidin was dissolved in ethanol, the effect of the solvent on SsLM was examined. QCM-D data show almost no shift in frequency or dissipation. Moreover, the membrane resistance did not decrease due to the addition of increasing amounts of ethanol and hence, pore formation induced by ethanol can be excluded. Noticeably, when adding increasing amounts of ethanol, the resistance of the lipid bilayer resting on the SbpA lattice was slightly increasing with time.

### 2.4. Reconstitution of α-Hemolysin

SsLMs were incubated with the exotoxin α-hemolysin dissolved in buffer at concentrations of 0.5, 0.75, and 1 µM (Figure 1, step 6B and Figure 2). Despite the importance of DMPE to bind vesicle on the SbpA lattice as mentioned above, DMPE is known to be unfavorable for α-hemolysin reconstitution. Therefore, the outer leaflet of the lipid bilayer of the SUVs was selectively modified by the insertion of DMPE. As previously demonstrated, the bilayer membrane of SUVs unfolds to expose the inner monolayer of the vesicles predominantly (at minimum 70%) to the bulk solution [46]. In turn, at least 70% of the membrane is expected to be oriented with the DMPE-enriched monolayer towards the SbpA lattice [46]. Importantly, the time required for lipids to be inserted only in the outer membrane must be taken into consideration. The typical time-scale for phospholipids to flip-flop from the outer to the inner monolayer is several days [46,47]. Thus, to be on the save side, liposomes were used immediately after incubation with DMPE for one hour at 4 °C.

QCM-D measurements showed during and after the incubation of SsLM with α-hemolysin no significant shifts of both, frequency and dissipation (Figure 2). Thus, one may conclude that the surface-attached mass comprising of biomolecules and water and the viscoelastic properties of the layer, respectively, remained constant. One explanation could be that there is no interaction between α-hemolysin and the SsLM in whatever way. Although not far too conceivable, in contrast, α-hemolysin may have been attached to the lipid surface and thus, caused a loss of acoustically sensed water due to the interaction of certain domains of α-hemolysin with lipid head groups. In addition, it has been shown, that at (planar) lipid bilayers approximately 20 water molecules are associated with each lipid molecule [48] and that an ordering of a layer several water molecules thick occurs [49,50]. Hence, in this scenario, the mass increase due to the attachment and possible aggregation of α-hemolysin on the lipid membrane is compensated by the loss in both, hydration of the lipid head groups and ordering the water layer. However, QCM-D experiments alone cannot provide any meaningful conclusion on the interaction of α-hemolysin with the SsLM.

To elucidate possible α-hemolysin-induced changes in the electrical properties of the SsLM, EIS experiments on the same specimen in the QCM-D flow cell have been performed. The obtained impedance results provide evidence for lipid attachment or aggregation of α-hemolysin and failure of pore formation in SsLM since the membrane resistance even increased from initially 6.96 to 10.75 MΩ·cm^2^ and 12.13 to 16.08 MΩ·cm^2^ before and after the addition of 0.5, 0.75, and 1 µM α-hemolysin, respectively. Note, as α-hemolysin is dissolved in buffer, no ethanol is present in these experiments, which might cause an increase of the membrane resistance. Indeed, SsLMs show high stability in terms of electrical tightness upon subsequent addition of up to 30 µM α-hemolysin. In order to provide a different lipid environment and hence, possibly an enhanced penetration activity of α-hemolysin, an alternative strategy was to replace Egg PC by 1,2-diphytanoyl-*sn*-glycerol-3-phosphatidyl choline (DPhPC). It has been proven that bilayers composed of Egg PC and DPhPC could be further stabilized by an attached SbpA lattice [22]. The difference in the composition of the two membrane architectures, but also whether cholesterol was present in the bilayer or not had no beneficial effect on the reconstitution of α-hemolysin. Despite the importance of cholesterol as a primary target for α-hemolysin, it makes protein insertion more difficult because cholesterol increases bilayer thickness and makes the lipid membrane more rigid [51,52]. An attempt was performed to incorporate α-hemolysin into SsLM (with Egg PC) without cholesterol but the obtained result did not improve significantly (data not shown).

Although the advantage of using S-layer proteins as a cushion or spacer layer providing a lubricant surface and enabling the self-healing of defects in supported membranes, the S-layer lattice may hinder the incorporation of proteins with bulky domains protruding the lipid membrane. In previous studies it has been demonstrated that α-hemolysin can be functional reconstituted into a free-standing lipid bilayer onto which an S-layer has previously been recrystallized [21,53]. The difference in the present study is that the S-layer lattice is with one face in contact with the solid support and with the other face with the head groups of the lipid membrane. In previous studies, however, the S-layer lattice was only in contact with the lipid head groups and the other face with the bulk water. Hence, it is conceivable that the structure of the S-layer lattice might be slightly modified at that portion, which interacts with the gold surface of the solid support and thus, α-hemolysin could not be reconstituted in its functional form. This assumption, however, has to be confirmed by additional surface-sensitive techniques and will be an objective of our further studies.

Reconstitution of α-hemolysin was previously investigated in alkane supported hybrid bilayer membranes [54]. The impedance model parameters suggested that α-hemolysin partially penetrates into the hybrid bilayer, whereby the dielectric constant of the alkane portion of the monolayer increased. The complete reconstitution of α-hemolysin, which could provide free access of ions to the metal surface, was not observed in this study [54].

Most recently, our preliminary data indicate the possibility to study the voltage-gated channel properties of the voltage dependent anion channel (VDAC) reconstituted in SsLM. Interestingly, despite both proteins, α-hemolysin and VDAC, have almost the same MW of approximately 33 kDa, the shape of the pore/channel, which is mushroom-like and cylindrical, respectively might affect whether functional incorporation may occur or not.

## 3. Experimental Section

### 3.1. Isolation of S-Layer Proteins

The bacterial cell surface layer protein SbpA (*M*w 127 kDa) was isolated from *Lysinibacillus sphaeri*cus CCM 2177 as previously described [55]. The protein solution (1 mg/mL) was diluted 1:10 prior use with crystallization buffer, 0.5 mM Tris-HCl, 10 mM CaCl_2_, pH 9 [14,18]. The self-assembly process on gold substrates is usually completed after 3 h. A subsequent rinsing step with water was applied in order to remove excess protein.

### 3.2. EDC Activation

SbpA lattice was modified with 1-ethyl-3-(3-dimethylaminopropyl) carbodiimide hydrochloride (EDC; Sigma-Aldrich, St. Louis, MO, USA) (15 mg/mL, pH 4.5). EDC reacts with the free carboxyl groups on the SbpA lattices and form highly reactive *O*-acylisourea intermediates [15]. In the following, vesicles containing DMPE were bound via EDC coupling onto the activated SbpA lattice [28].

### 3.3. Vesicle Preparation

Egg yolk phosphatidyl choline (Egg PC) and 1,2-dimyristoyl-*sn*-glycero-3-phospho-ethanolamine (DMPE) were purchased from Sigma-Aldrich. Cholesterol and 1,2-diphytanoyl-*sn*-glycerol-3-phosphatidyl choline (DPhPC) were purchased from Avanti Polar Lipids Inc. (Alabaster, AL, USA). The β-diketon ligand has been synthesized as described elsewhere [56].

Two strategies have been followed to prepare liposomes. First strategy: Egg PC: DMPE: cholesterol (molar ratio 4:1:1) and 1% β-diketone ligand were dissolved in chloroform in a round bottom flask, dried under vacuum for at least 3 h at 45 °C. The lipid film was then rehydrated in 200 mM sucrose and extruded 21 times through a 100 nm polycarbonate membrane (Whatman, Kent, UK) by using a Mini Extruder (Avanti Polar Lipid Inc., Alabaster, AL, USA) to form a suspension of small unilamellar vesicles (SUVs). Second strategy: Egg PC or DPhPC: cholesterol (molar ratio 4:1) and 1% β-diketone ligand were dissolved in chloroform, dried under vacuum for at least 3 h at 45 °C. The lipid film then was rehydrated and extruded as described above. To selectively modify the outer lipid monolayer of the vesicles, 30 µg/mL DMPE was dissolved in chloroform and dried for at least 3 h at room temperature. After solubilizing the lipid with a small quantity of ethanol (~15 µL), the previously prepared liposomes were added and incubated for one hour at 4 °C. Before use, vesicles no matter which strategy was used were diluted in sucrose/glucose (1/2) to give a final lipid concentration of 1 mg/mL.

*3.4. α-Hemolysin and Gramicidin*

α-Hemolysin (No. H9395) and gramicidin (No. 50845) were purchased from Sigma-Aldrich. A stock solution of 0.5 mg/mL α-hemolysin in 10 mM HEPES, 150 mM NaCl buffer, pH 7.4, was prepared and stored at 4 °C [34]. Before use, this stock solution was diluted with the same buffer to obtain the desired concentrations (0.50, 0.75 and 1 µM, respectively).

Since gramicidin is almost insoluble in water, a stock solution of 1 mg/mL in ethanol was prepared and stored at 4 °C. The desired concentrations (1, 2.5 and 5 µM) were diluted in 10 mM HEPES, 150 mM NaCl buffer, pH 7.4, before injection into QCM-D chamber. It is expected that the concentration of gramicidin in the lipid bilayer is lower than the added amount due to the limited solubility of gramicidin in the aqueous phase.

### 3.5. Quartz Crystal Microbalance with Dissipation Measurement (QCM-D)

Commercially available sensor crystals, 14 mm in diameter and coated with a 100 nm thick gold layer (QSX 301; Q-Sense AB, Västra Frölunda, Sweden), suitable for the QCM-D E4 device (Q-Sense AB) were used in the present study. The measured signals by this technique are the change in frequency and dissipation as a function of time. A shift in frequency is related to the mass of the material adsorbed on or removed from the sensor surface, while a shift in dissipation provides information on the viscoelastic properties of the adsorbed materials before, during, and after the interaction. QCM-D experiments were measured at 25 ± 0.02 °C. The flow rate for SbpA injection was 50 and 25 μL/min for all others fluid injections and rinsing steps, respectively. QCM-D data were recorded from the 1^st^ to the 13^th^ odd-numbered overtones; the data, e.g., in Figure 2 are shown for the 7^th^ overtone.

### 3.6. Electrochemical Impedance Spectroscopy (EIS)

EIS measurements were performed using the Q-sense Electrochemistry Module (QEM 401; Q-Sense AB) connected to a potentiostat (CHI660c; CH Instrument, Austin, TX, USA). For electrochemical measurements, the QCM-D sensor was used as working electrode combined with a platinum counter-electrode on the top wall of the chamber and an Ag/AgCl reference electrode, which is fixed in the outlet flow channel. Measurements were performed in the frequency range of 100 mHz to 100 kHz. An AC potential of 15 mV was applied at a DC voltage of 0 mV *versus* the reference electrode. The electrolyte used in all experiments was 10 mM HEPES buffer, 150 mM NaCl, pH 7.4.

All impedance data were fitted by the CHI software in a parallel equivalent circuit (*R_el_* − *R_m_*) × *Z*_CPE_; where *R_el_* is the electrode resistance, *R_m_* is the membrane resistance, and *Z*_CPE_ is the impedance of the constant phase element (CPE) [57]. Using CPE instead of the ideal membrane capacitance improve the quality of the fit significantly [58,59]. However, from the CPE value and by estimation of a dimensionless parameter (between zero and one) it is possible to deduce the membrane capacitance [59]. The surface area specific electrolyte resistance was determined to be between 5 and 7 Ω·cm^2^ in all performed measurements.

## 4. Conclusions

In summary, we have reported on the generation and performance of a biomimetic platform, the SsLM, which accomplished an electrical sealing feasible to examine the ion flux through incorporated membrane functions. As no aperture is necessary to generate the SsLM in the QCM-D cell, combined QCM-D and EIS measurements can be performed on one and the same SsLM preparation. QCM-D measurements quantify the recrystallized and adsorbed materials on the surface while EIS confirms ions flux through the SsLM and formation of functional pores or channels. The present SsLM platform offers many advantages for the ionophore gramicidin. Indeed, the concentration dependent ion flux through the gramicidin channels incorporated in the SsLM could be measured by EIS. However, reconstitution of the transmembrane protein α-hemolysin could not be achieved since the impedance results provide evidence for lipid attachment or aggregation of α-hemolysin but failure of pore formation in SsLM because the membrane resistance even increased after each addition of α-hemolysin. Hence, the reconstitution of large proteins is still limited and seems to be governed by many factors such as the shape of the pore/channel, the nature of the lipid molecules and their interaction with membrane proteins, the structure of the tethering structure, *etc.* The SsLM, however, might be a versatile lipid membrane platform suitable to elucidate these critical factors for transmembrane protein reconstitution in particular as many (combined) surface-sensitive techniques can be applied.

In future, increased knowledge of the requirements for membrane protein reconstitution might help to think over rebuilding even sensory organs, for example artificial noses or tongues, based on this biomimetic platform where many receptor proteins have to be exposed and read out simultaneously. Finally, planar model lipid membranes in general and SsLMs in particular are of high interest for the development of versatile biosensors based on membrane-active peptides and membrane proteins used as responsive elements.
